# External validation and comparison of five prediction models for lymph node metastasis after noncurative endoscopic submucosal dissection for early gastric cancer

**DOI:** 10.1007/s10120-026-01798-9

**Published:** 2026-09-07

**Authors:** Ayaka Takasu, Waku Hatta, Takuji Gotoda, Yohei Ogata, Yuichiro Kemmoto, Hiroto Noda, Souya Nunobe, João Santos-Antunes, Hiroshi Kawachi

**Affiliations:** 1 Department of Gastroenterology, Cancer Institute Hospital, Japanese Foundation for Cancer Research, Tokyo, Japan; 2 Division of Pathology, Cancer Institute, Japanese Foundation for Cancer Research, Tokyo, Japan; 3 Division of Gastroenterology and Hepatology, Department of Medicine, Nihon University School of Medicine, Tokyo, Japan; 4 Division of Gastroenterology, Tohoku University Graduate School of Medicine, 1-1 Seiryo-Machi, Aoba-Ku, Sendai, Miyagi 980-8574 Japan; 5 Department of Gastroenterology, Nippon Medical School Hospital, Tokyo, Japan; 6 Department of Gastroenterological Surgery, Cancer Institute Hospital, Japanese Foundation for Cancer Research, Tokyo, Japan; 7 Department of Gastroenterology, Centro Hospitalar Universitário de São João, Porto, Portugal; 8 Department of Pathology, Cancer Institute Hospital, Japanese Foundation for Cancer Research, Tokyo, Japan

**Keywords:** Stomach neoplasms, Endoscopic submucosal dissection, Lymphatic metastasis

## Abstract

**Background:**

Additional gastrectomy is recommended after noncurative endoscopic submucosal dissection (ESD) for early gastric cancer (EGC) despite the low incidence of lymph node metastasis (LNM). The eCura system, W-eCura score, and 11-point score reportedly predict LNM risk; however, their comparative performance remains unclear. We aimed to externally validate and directly compare five risk stratification approaches in patients with noncurative ESD.

**Methods:**

We retrospectively evaluated 634 consecutive patients with EGC who underwent noncurative ESD at a single high-volume center. Five prediction models were evaluated: the eCura system (7-point score and three-risk classification), W-eCura score (7-point score and three-risk classification), and 11-point score. All pathological findings were re-evaluated by an outcome-blinded expert pathologist. Model performance was assessed by discrimination, calibration, and decision-curve analysis (DCA).

**Results:**

LNM was identified in 26 (4.9%) patients. All models showed comparable discrimination (c-statistics, 0.71 to 0.72). However, calibration-in-the-large and calibration slope were poorer in the W-eCura score and 11-point score (− 1.43 to − 0.96 and 0.26 to 0.64) than in the eCura system (− 0.43 to − 0.40 and 0.83 to 0.84). Using DCA, the eCura system and W-eCura score consistently showed higher net benefits than did the 11-point score across clinically relevant threshold probabilities. Recalibration improved both calibration and net benefits across all models.

**Conclusions:**

Despite similar discriminative performance, the eCura system demonstrated relatively favorable calibration and potential clinical utility in this Japanese cohort. Given their simplicity and structural similarity, three-risk classifications of the eCura system and W-eCura score, when appropriately calibrated, may support decision-making regarding additional gastrectomy.

**Supplementary Information:**

The online version contains supplementary material available at https://doi.org/10.1007/s10120-026-01798-9.

## Introduction

Endoscopic submucosal dissection (ESD) is widely accepted as a minimally invasive and curative treatment of early gastric cancer (EGC) with an extremely low risk of lymph node metastasis (LNM) [1, 2]. However, when pathological findings indicate noncurative resection, additional gastrectomy with lymph node dissection is recommended according to the European and Japanese guidelines [3, 4]. Nevertheless, the incidence of LNM in patients with noncurative ESD is reported to be approximately 5% to 10%, suggesting that additional surgery might represent overtreatment in a substantial proportion of patients [5–8].

Several predictive models have been proposed to stratify LNM risk after noncurative ESD. The eCura system is a 7-point scoring system with three-risk classifications based on five pathological factors: lymphatic invasion, tumor size, depth of submucosal invasion, venous invasion, and vertical margin status [9]. The W-eCura score modifies the cutoff value for submucosal invasion depth based on Western data [10]. Another model, the 11-point score, has been proposed based on pathological factors such as tumor size, invasion depth, histological subtype (including mixed-type histology), ulcerative findings, and lymphovascular invasion [11].

Despite the availability of these prediction models, it remains unclear which model provides the most appropriate risk stratification in clinical settings. A recent retrospective multicenter study has compared the performance between the eCura system and W-eCura score [12]; however, this study has several limitations, including the use of only the three-risk classification for model comparison, potential variability in pathological assessment across observers and institutions, and missing data of several pathological variables [13]. Additionally, the proportion of patients who underwent additional surgery was not reported, potentially introducing a selection bias [13]. Therefore, further validation in a well-defined cohort with standardized pathological evaluation is warranted.

The aim of this study was to externally validate and directly compare five risk stratification approaches, the 7-point score and three-risk classification of the eCura system and W-eCura score and the 11-point score, in a consecutive cohort of patients who underwent noncurative ESD, with systematic pathological re-evaluation.

## Methods

### Study design and patients

This retrospective observational study was conducted at a single high-volume center to externally validate five LNM prediction models in patients with EGC after noncurative ESD: the eCura system, W-eCura score (each evaluated as both a 7-point score and a three-risk classification), and 11-point score (Supplementary Table 1).

We screened consecutive patients with EGC who underwent endoscopic resection at the Cancer Institute Hospital of the Japanese Foundation for Cancer Research between January 2011 and March 2025. Patients initially classified as having noncurative resection based on the original pathological diagnosis, as defined according to the Japanese Gastric Cancer Treatment Guidelines, were identified [4]. Data of patients who underwent additional surgical resection with lymph node dissection and had a pathologically evaluable LNM status were included in the analysis. Patients managed with surveillance were excluded because the pathological confirmation of LNM was unavailable. Clinical information, endoscopic findings, and surgical data were retrospectively collected from the electronic medical records.

The exclusion criteria were as follows:

(1) synchronous EGC that did not meet the curative criteria for ESD; (2) synchronous or metachronous advanced gastric cancer; (3) endoscopic resection performed in the remnant stomach; (4) additional treatment other than gastrectomy; (5) invasion of the muscularis propria or deeper identified in the surgical specimen; (6) patients with other active malignancies that precluded treatment decisions based on oncological factors; and (7) reclassification as curative resection after expert pathological re-evaluation.

### Pathological evaluation

Pathological diagnoses were made according to the Japanese Classification of Gastric Carcinoma issued by the Japanese Gastric Cancer Association (JGCA) [14]. Endoscopic resection specimens were re-evaluated with a focus on variables included in the LNM prediction models, such as the tumor size, depth of submucosal invasion, ulcerative findings (defined as a peptic ulcer or ulcer scar), lymphatic invasion, venous invasion, vertical margin status, and histological type. Pathological re-evaluation was performed using hematoxylin and eosin- and periodic acid–Schiff-stained sections, which were available for all tumor-containing sections in all cases, as well as additional stained sections obtained during the routine pathological assessment. Additional staining was also routinely performed on representative sections in all submucosal cancers, irrespective of the histological findings, and in selected mucosal cancers with specific pathological findings, such as invasion into the muscularis mucosae or extension of a poorly differentiated adenocarcinoma component into the deep lamina propria. These additional stains included D2-40 immunohistochemistry for the assessment of lymphatic invasion; elastic fiber staining with Victoria blue or Elastica van Gieson for the assessment of venous invasion; and immunohistochemical staining for CD31/platelet endothelial cell adhesion molecule−1, desmin, and cytokeratin AE1/AE3. The institutional routine for elastic fiber staining changed from Victoria blue to Elastica van Gieson during 2019.

Regarding histological type, the classification system of the Japanese Classification of Gastric Carcinoma, which differs slightly in terminology, is fundamentally identical in framework to that of the World Health Organization (WHO), allowing for complete correspondence between the two. Specifically, JGCA categories pap, tub1, and tub2 correspond to the WHO categories of papillary and tubular (well- and moderately differentiated) types, whereas JGCA categories por and sig correspond to WHO tubular (poorly differentiated) and poorly cohesive types [15]. In this study, to enable more appropriate comparisons with previous studies, we adopted the WHO classification terminology and divided differentiation into two groups: “well/moderate” and “poor” [10]. However, tumors containing both well/moderate and poor components were defined as mixed type and were further subclassified on the basis of the predominant component into mixed type, well/moderate predominant, mixed type, and poor predominant. Regarding the risk score calculation, histological classification was based on the predominant component of the eCura system and W-eCura score, whereas for the 11-point score, the mixed histological categories, described earlier, were applied. Re-evaluation was performed by an expert gastrointestinal pathologist who was blinded to the original pathological diagnosis, clinical information, and outcome data, including LNM.

### Risk score assessment

In each patient, risk scores were calculated on the basis of the pathological findings of the endoscopic resection specimens according to the original definitions of the eCura system, W-eCura score, and 11-point score. The eCura system assigns points for lymphatic invasion (3 points), tumor size >30 mm (1 point), submucosal invasion ≥500 μm (1 point), venous invasion (1 point), and a positive vertical margin (1 point). Patients were evaluated on the basis of both the eCura system (7-point score) and eCura system (three-risk classification). For the three-risk classification, patients were categorized as low-risk (0–1), intermediate-risk (2–4), and high-risk (5–7). The W-eCura score uses the same variables as the eCura system but applies a different cutoff value for submucosal invasion (≥ 1000 μm). The patients were evaluated on the basis of both the W-eCura score (7-point score) and corresponding three-risk classification, categorized as low-risk (0–1), intermediate-risk (2–4), or high-risk (5–7). Category-specific LNM rates for the W-eCura 7-point score, which were not reported in the original publication, were obtained from the original authors. The 11-point score assigns points on the basis of lymphatic or venous invasion (4 points), undifferentiated-predominant (poor predominant) mixed type (2 points), submucosal invasion ≥ 500 μm (2 points), tumor size >30 mm (2 points), differentiated-predominant (well/moderate predominant) mixed type (1 point), pure undifferentiated (pure poor) type (1 point), tumor size > 20 mm and ≤ 30 mm (1 point), and ulcerative findings (1 point) (Supplementary Table 1). For risk score calculation, cases with an unclear vertical margin were classified as having a positive vertical margin.

### Outcome

The primary outcome was LNM, which was defined as pathological evidence of metastasis in the lymph nodes retrieved during additional surgical resection.

### Statistical analysis

Continuous variables are presented as median (interquartile range), and categorical variables are presented as number and percentages. The predictive performance of each risk score was evaluated by assessing discrimination and calibration. Discrimination was quantified by the concordance statistic (c-statistic); receiver operating characteristic curves were generated to visually compare the discriminatory performance. Discrimination was considered acceptable when the c-statistic was ≥ 0.70. Calibration was performed using calibration plots, calibration-in-the-large plots, and slopes. Overall performance was assessed by the Brier score. Calibration-in-the-large indicates whether the predicted risks are systematically over- or underestimated, with a value of zero representing perfect agreement between the predicted and observed risks. The calibration slope reflects the agreement between the predicted and observed risks, with a value of 1 indicating perfect calibration, values < 1 indicating overfitting, and values > 1 indicating underfitting. The Brier score ranges between 0 and 0.25 for binary outcomes, with 0 indicating perfect prediction and lower values indicating better overall predictive accuracy. Recalibration was performed using logistic recalibration by simultaneously updating the model intercept and calibration slope. The decision-curve analysis (DCA) was performed to evaluate the net clinical benefit of each model across a range of threshold probabilities. All statistical analyses were performed using SPSS Statistics (version 31; IBM Corp., Armonk, NY, USA) and R software (version 4.4.3; R Foundation for Statistical Computing, Vienna, Austria).

### Ethics statements

This study was approved by the Institutional Review Board of the Cancer Institute Hospital of the Japanese Foundation for Cancer Research (approval number: 2025-GB-039). The requirement for informed consent was waived due to using an opt-out approach.

## Results

### Patient characteristics and incidence of lymph node metastasis

**Fig. 1:**
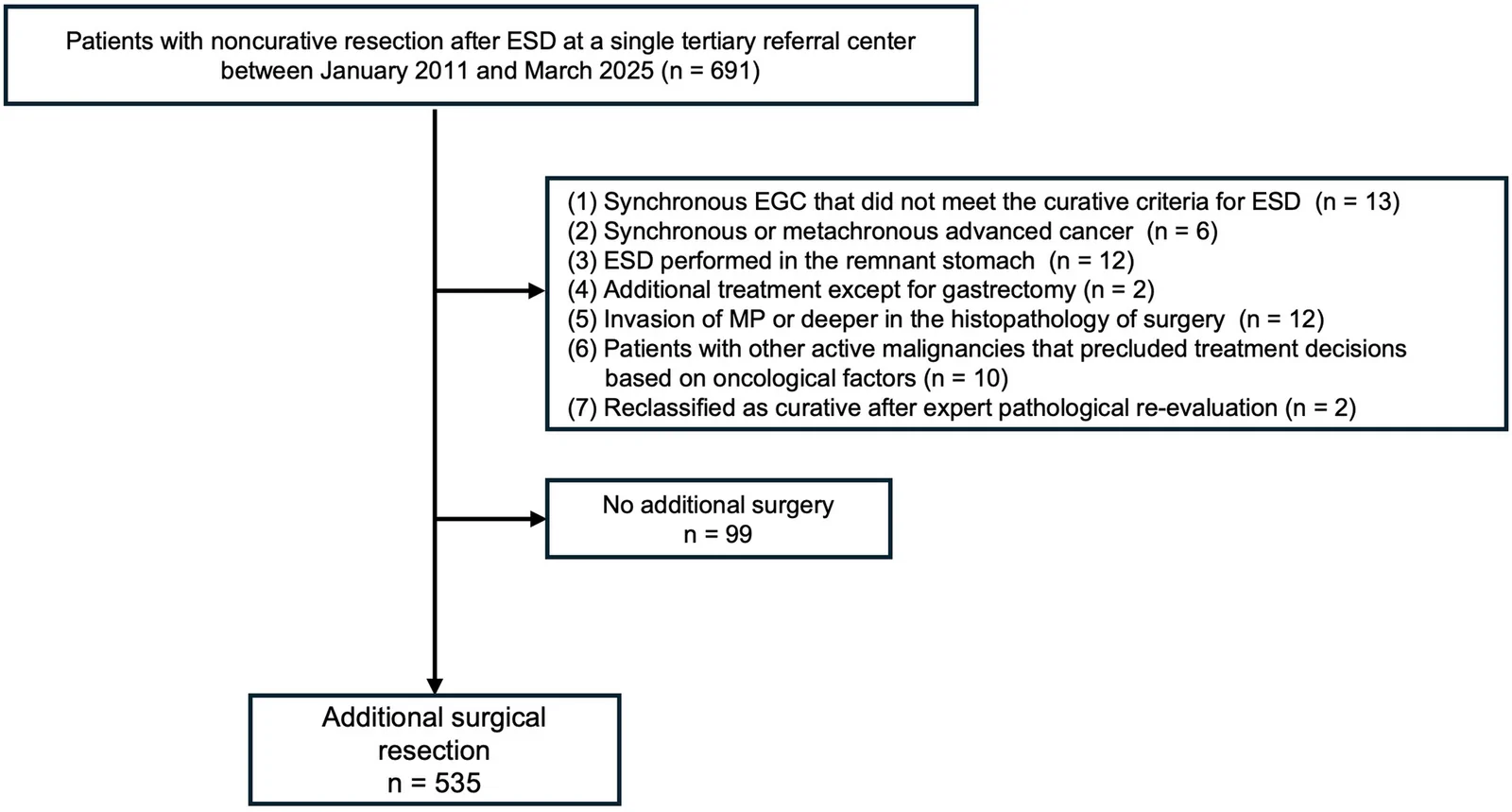
Study flow diagram. Among the 691 patients initially classified as having noncurative resection after ESD based on the original pathological diagnosis between January 2011 and March 2025, 57 were excluded according to the predefined criteria, including 2 patients who were reclassified as curative after expert pathological re-evaluation. Of the remaining 634 patients, 535 underwent additional gastrectomy with lymph node dissection and were included in the primary analysis, while 99 were managed with surveillance and excluded from the analysis. EGC, early gastric cancer; ESD, endoscopic submucosal dissection; MP, muscularis propria

Between January 2011 and March 2025, 691 patients were initially classified as having noncurative resection after ESD based on the original pathological diagnosis. Of these, 57 were excluded according to the predefined exclusion criteria, including two patients who were reclassified as curative after expert pathological re-evaluation; eventually, the data of 634 patients were used for the analysis (Fig. 1). Among the 634 patients, 535 (84.4%) underwent additional gastrectomy with lymph node dissection and were included in the study, whereas 99 (15.6%) underwent surveillance. LNM was pathologically confirmed in 26 of the 535 patients (4.9%). Baseline clinicopathological characteristics of the study population are summarized in Table 1.

**Table 1:** Baseline clinicopathological characteristics of the patients who underwent additional surgery after noncurative ESD

Variable^a^	n = 535
Age (years), median [IQR]	68.0 [59.0 to 74.0]
Sex	
Male	299 (55.9)
Female	236 (44.1)
Location, n (%)	
Upper third	115 (21.5)
Middle third	247 (46.2)
Lower third	173 (32.3)
Tumor size (mm), median [IQR]	22.0 [15.0 to 31.0]
Invasion depth, n (%)	
Mucosa	143 (26.7)
Submucosal invasion depth <500 µm	156 (29.2)
Submucosal invasion depth ≥500 µm	236 (44.1)
Submucosal invasion depth ≥1000 µm	115 (21.5)
Tumor differentiation, n (%)	
Pure type, well/moderate	172 (32.1)
Pure type, poor	78 (14.6)
Mixed type, well/moderate predominant	188 (35.1)
Mixed type, poor predominant	97 (18.1)
Lymphatic invasion, n (%)	
Negative	328 (61.3)
Positive	207 (38.7)
Venous invasion, n (%)	
Negative	384 (71.8)
Positive	151 (28.2)
Ulcerative finding^b^, n (%)	
Negative	466 (87.6)
Positive	66 (12.4)
Vertical margin, n (%)	
Negative	470 (87.9)
Positive	37 (6.9)
Unclear	28(5.2)
Lymph node metastasis, n (%)	26 (4.9)
Residual tumor, n (%)	4 (0.7)

^a^Values are presented as median (IQR) or number (%)

^b^Ulcerative findings indicated the presence of peptic ulcers or scars

*ESD* endoscopic submucosal dissection, *IQR* interquartile range

### Risk stratification and LNM incidence across the prediction models

Regarding both the eCura system (7-point score) and W-eCura score (7-point score), the incidence of LNM increased stepwise with increasing score values. Using the eCura system (three-risk classification), the incidences of LNM were 1.6%, 5.6%, and 13.0% in the low-, intermediate-, and high-risk groups, respectively. A similar stepwise increase was found using the W-eCura score (three-risk classification). Using the 11-point score, the incidence of LNM increased with higher scores (Supplementary Table 2). Among the 535 patients included in the primary analysis, 120 (22.4%) had a submucosal invasion depth of 500 to 999 μm, of whom 6 (5.0%) had LNM. Among these patients, risk classification differed between the eCura system and W-eCura score in 63 patients (Supplementary Table 3).

### Comparison of discrimination performance among the prediction models

**Fig. 2:**
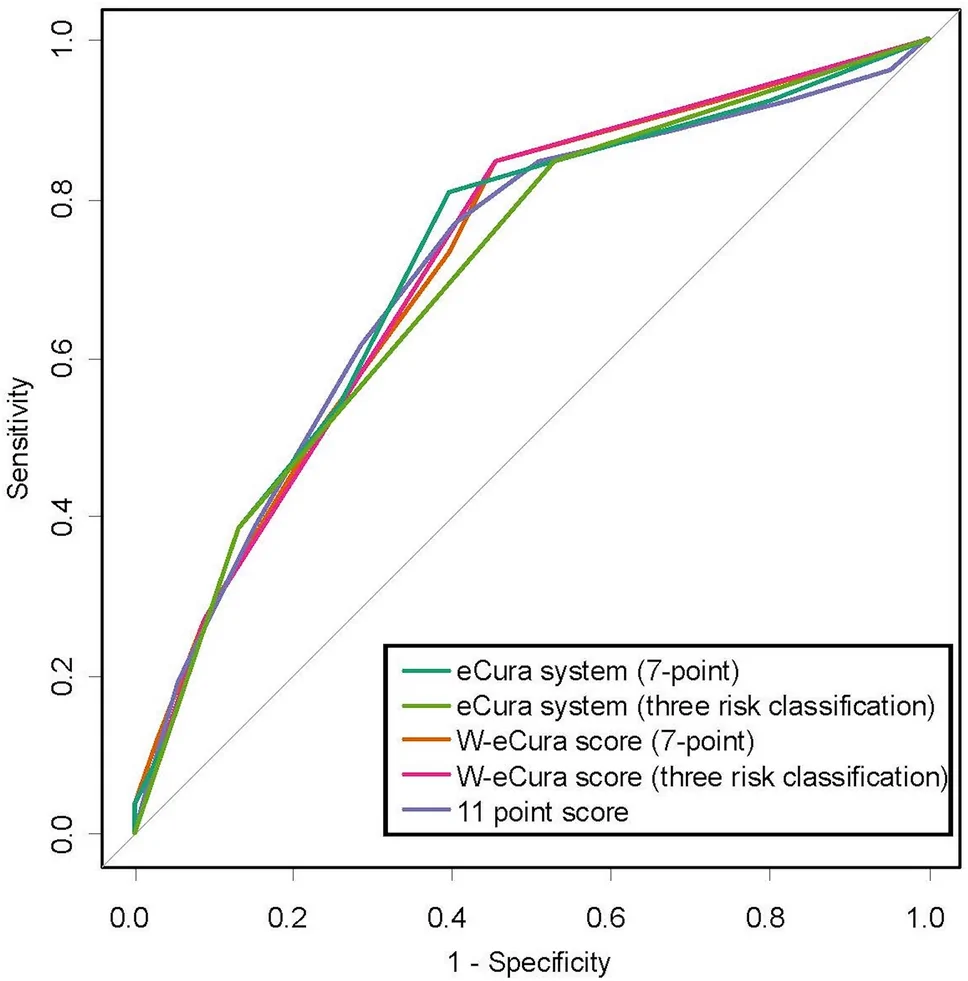
ROC curves of the five prediction models for LNM after noncurative ESD. The curves represent the eCura system (7-point score), eCura system (three-risk classification), W-eCura score (7-point score), W-eCura score (three-risk classification), and 11-point score. The diagonal line indicates the line of no discrimination. ROC, receiver operating characteristic; LNM, lymph node metastasis; ESD, endoscopic submucosal dissection

The discriminatory performance of each prediction model was assessed using c-statistics. The c-statistics were 0.72 (95% confidence interval [CI]: 0.62 to 0.82) for the eCura system (7-point score), 0.71 (95% CI: 0.61 to 0.81) for the eCura system (three-risk classification), 0.72 (95% CI: 0.63 to 0.82) for the W-eCura score (7-point score), 0.72 (95% CI: 0.63 to 0.81) for the W-eCura score (three-risk classification), and 0.71 (95% CI: 0.61 to 0.82) for the 11-point score. Discrimination was comparable across all the models (Fig. 2, Table 2).

**Table 2:** Performance of the five prediction models for LNM in patients who underwent additional surgery after noncurative ESD

Prediction model^a^	c-statistic	Calibration-in-the-large^b^	Calibration slope^c^	Brier score^d^
eCura system (7-point score)	0.72 (0.62 to 0.82)	− 0.40 (− 0.81 to 0.003)	0.83 (0.41 to 1.25)	0.05 (0.03 to 0.06)
eCura system (three-risk classification)	0.71 (0.61 to 0.80)	− 0.43 (− 0.83 to − 0.02)	0.84 (0.41 to 1.27)	0.05 (0.03 to 0.06)
W-eCura score (7-point score)	0.72 (0.60 to .82)	− 1.16 (− 1.61 to − 0.71)	0.26 (0.11 to 0.40)	0.07 (0.05 to 0.09)
W-eCura score (three-risk classification)	0.72 (0.63 to 0.81)	− 0.96 (− 1.41 to − 0.52)	0.33 (0.16 to 0.50)	0.06 (0.05 to 0.08)
11-point score	0.71 (0.61 to 0.82)	− 1.43 (− 1.84 to − 1.03)	0.64 (0.26 to 1.02)	0.07 (0.06 to 0.08)

*LNM* lymph node metastasis, *ESD* endoscopic submucosal dissection, *CI* confidence interval

^a^Values are presented as c-statistics, calibration-in-the-large, calibration slope, and Brier scores with 95% confidence intervals (CIs)

^b^Calibration-in-the-large reflects the systematic over- or under-estimation of the predicted risk, with values close to zero indicating good calibration

^c^The calibration slope assessed the agreement between the predicted and observed risks, with a value of 1 indicating perfect calibration

^d^The Brier score ranges from 0 to 0.25, with 0 indicating perfect prediction, and lower values indicating better overall predictive accuracy

### Comparison of calibration and predictive accuracy

**Fig. 3:**
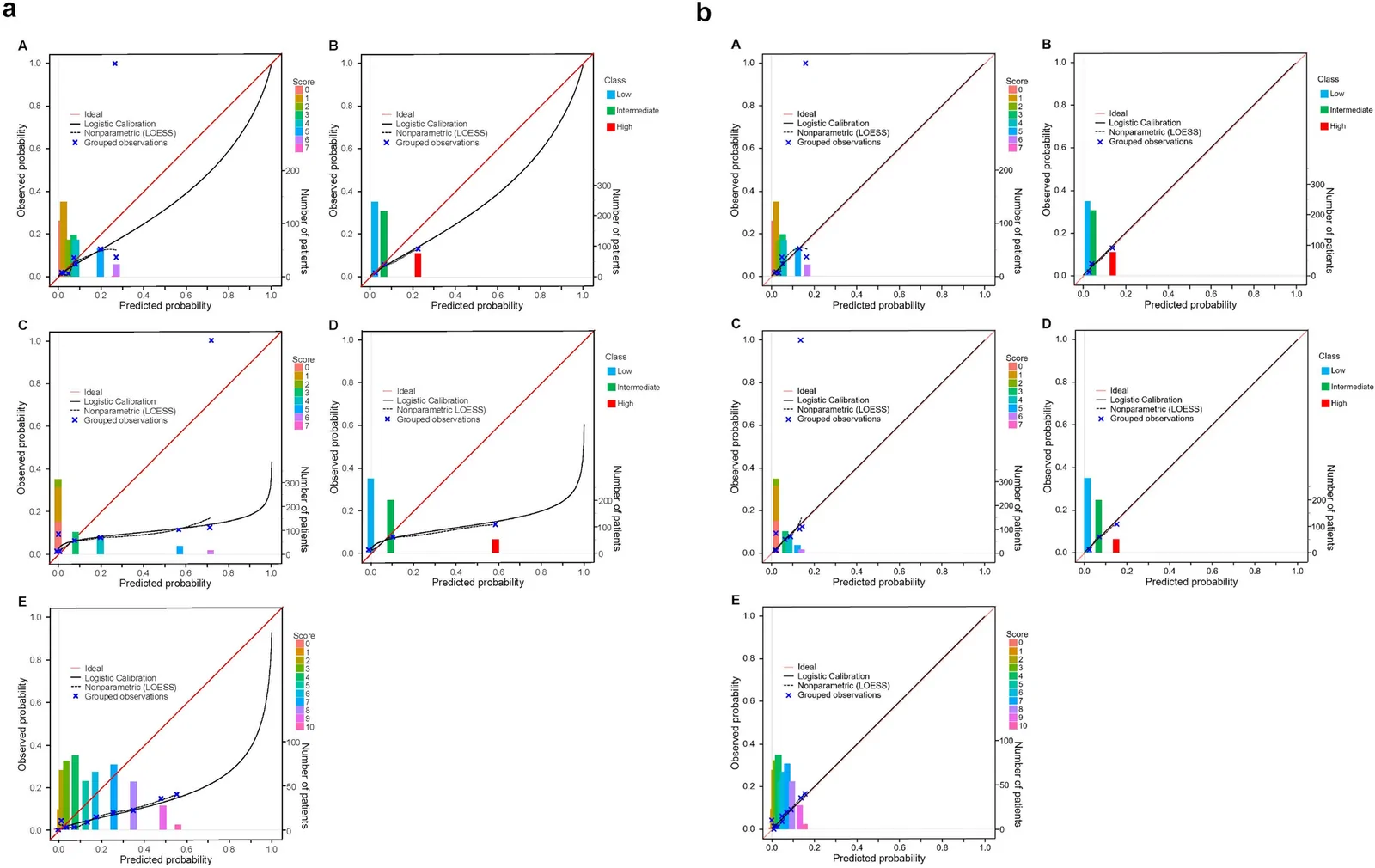
Calibration plots of the five prediction models for LNM after noncurative ESD before and after recalibration. **a** Original calibration plots of the five prediction models. **b** Calibration plots of the five prediction models after recalibration. **A** eCura system (7-point score), **B** eCura system (three-risk classification), **C** W-eCura score (7-point score), **D** W-eCura score (three-risk classification), **E** 11-point score. The red line represents the ideal calibration. The solid black line indicates the logistic calibration, and the dashed black line represents the nonparametric calibration estimated by LOESS. The blue crosses indicate grouped observations. Histograms show the distribution of the predicted probabilities. LNM, lymph node metastasis; ESD, endoscopic submucosal dissection; LOESS, locally weighted scatterplot smoothing

The calibration performance of each prediction model was evaluated using calibration plots, calibration-in-the-large slopes, and Brier scores (Fig. 3a, Table 2). Calibration-in-the-large values were −0.40 (95% CI: − 0.81 to 0.003) and − 0.43 (95% CI: − 0.83 to − 0.02) for the eCura system (7-point score and three-risk classification), − 1.16 (95% CI: − 1.61 to − 0.71) and − 0.96 (95% CI: − 1.41 to − 0.52) for the W-eCura score, and − 1.43 (95% CI: − 1.84 to − 1.03) for the 11-point score, indicating greater overestimation of risk in the latter models. Calibration slopes were higher for the eCura system (7-point score: 0.83; three-risk classification: 0.84) than for the W-eCura score (7-point score: 0.26; three-risk classification: 0.33) and 11-point score (0.64). The Brier scores were 0.05 for both the eCura system (7-point score and three-risk classification), 0.07 and 0.06 for the W-eCura score (7-point score and three-risk classification), and 0.07 for the 11-point score, indicating similar prediction accuracy across the models.

### Model performance after recalibration

After recalibration, the calibration-in-the-large values approached zero, and the calibration slopes approached one for all the models (Fig. 3b, Supplementary Table 4). The Brier scores also became similar across the models, indicating improved overall calibration performance.

### Decision-curve analysis

**Fig. 4:**
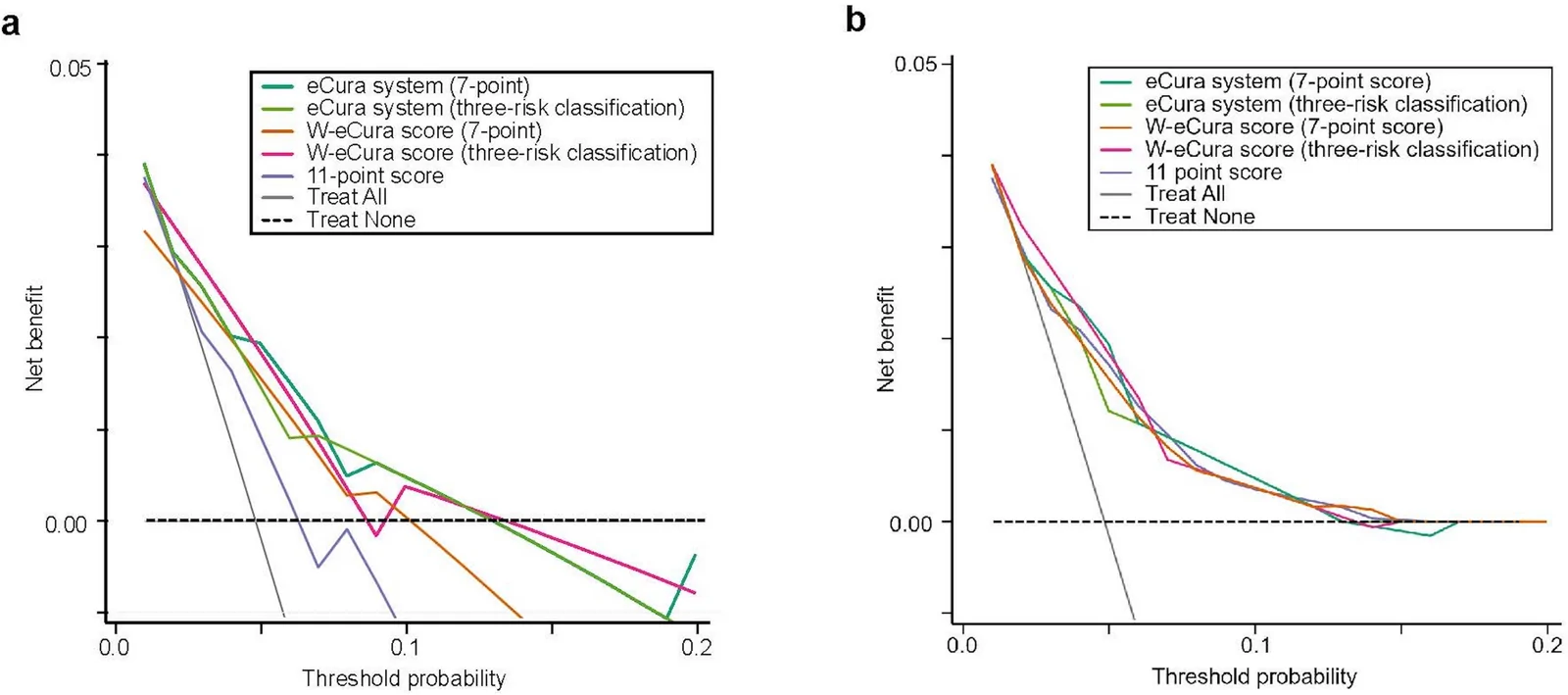
DCA of the five prediction models for LNM after noncurative ESD before and after recalibration. **a** Original DCA of the five prediction models. **b** DCA of the five prediction models after recalibration. The curves represent the eCura system (7-point score), eCura system (three-risk classification), W-eCura score (7-point score), W-eCura score (three-risk classification), and 11-point score. The x-axis represents the threshold probability, and the y-axis represents the net benefit. The gray line indicates the strategy for treating all patients, whereas the dashed line indicates no treatment. A higher net benefit indicates greater clinical utility. DCA, decision-curve analysis; LNM, lymph node metastasis; ESD, endoscopic submucosal dissection

Using the DCA, the eCura system and W-eCura score (7-point score and three-risk classification) demonstrated positive net benefit across clinically relevant ranges of threshold probabilities (0.02–0.13 for the eCura system and 0.02–0.10 for the W-eCura score) (Fig. 4a). Both models consistently showed a net benefit higher than the 11-point score across most threshold probabilities. By contrast, the 11-point score showed a narrower range of clinical utility, with a net benefit of only over a narrow threshold range (0.02–0.06). After recalibration, the net benefit curves of all models were similar, with minimal differences observed across the threshold probabilities (Fig. 4b).

### Subgroup analysis by the histological type

The subgroup analyses were performed according to the histological type, dividing the patients into well/moderate and poor groups. Regarding the eCura system, the c-statistic values were 0.71 (95% CI: 0.59 to 0.83) and 0.75 (95% CI: 0.56 to 0.94) for the 7-point score, and 0.70 (95% CI: 0.58 to 0.82) and 0.72 (95% CI: 0.52 to 0.92) for the three-risk classification, in the well/moderate and poor groups, respectively. Considering the W-eCura score, the corresponding values were 0.70 (95% CI: 0.58 to 0.81) and 0.77 (95% CI: 0.60 to 0.95) for the 7-point score, and 0.70 (95% CI: 0.59 to 0.81) and 0.75 (95% CI: 0.55 to 0.95) for the three-risk classification. For the 11-point score, the c-statistic values were 0.73 (95% CI: 0.61 to 0.85) and 0.70 (95% CI: 0.52 to 0.89), respectively (Table 3).

**Table 3:** Discrimination of five prediction models according to histological type after noncurative ESD

Prediction model^a^	c-statistic (95% CI)	
	Well/moderate group (n = 360)	Poor group (n = 175)
eCura system (7-point score)	0.71 (0.59 to 0.83)	0.75 (0.56 to 0.94)
eCura system (three-risk classification)	0.70 (0.58 to 0.82)	0.72 (0.52 to 0.92)
W-eCura score (7-point score)	0.70 (0.58 to 0.81)	0.77 (0.60 to 0.95)
W-eCura score (three-risk classification)	0.70 (0.59 to 0.81)	0.75 (0.55 to 0.95)
11-point risk score	0.73 (0.61 to 0.85)	0.70 (0.52 to 0.89)

*ESD* endoscopic submucosal dissection, *CI* confidence interval

^a^Values are presented as c-statistics with 95% CIs

## Discussion

In this study, we externally validated five LNM prediction models: the eCura system (7-point score), eCura system (three-risk classification), W-eCura score (7-point score), W-eCura score (three-risk classification), and 11-point score, in a single cohort of patients with EGC after noncurative ESD. Although all models showed comparable discrimination, important insights into their clinical utility were obtained through the evaluation of calibration and DCA.

All models demonstrated similar discrimination, with a c-statistic of approximately 0.70. The eCura system and W-eCura score shared the same major predictors except for the cutoff value of submucosal invasion (500 μm versus [vs.] 1000 μm), and thus their comparable discrimination is expected. By contrast, despite having a more granular and complex scoring structure and requiring eight parameters to assess the same five pathological factors, the 11-point score did not demonstrate improved discrimination over the simpler eCura system and W-eCura score. These findings suggest that increasing the model complexity would not necessarily lead to improved predictive performance in terms of discrimination.

In contrast to discrimination, calibration differed across the models. The W-eCura and 11-point scores showed poorer calibration than did the eCura system, with a tendency to overestimate the LNM risk. This miscalibration possibly reflected differences in the baseline LNM incidence between the cohorts, particularly for the W-eCura score, which was developed in Western populations with distinct cohort characteristics, including pathological assessment and specimen processing, rather than fundamental differences in the model structure. The eCura system, developed in a Japanese cohort, was more consistent with the present study population. These background differences might have contributed to the observed differences in the calibration. Notably, a recent multicenter Japanese validation study by Yabuuchi et al. demonstrated good calibration of the eCura system and a stepwise increase in the LNM incidence across the risk categories [12]. Together with our findings, this suggests that although the eCura system may provide consistent risk stratification across cohorts, absolute risk estimates may vary according to cohort characteristics. Therefore, calibration should be evaluated in the target population, and recalibration may be necessary when applying the model to different clinical settings. Another potential explanation is the difference in the cutoff value for submucosal invasion. In our cohort, 120 patients (22.4%) had a submucosal invasion depth of 500 to 999 μm, with LNM observed in 6 patients (5.0%). Because these patients receive an additional point in the eCura system but not in the W-eCura score, risk classification differed between the two models in 63 patients (Supplementary Table 3). These differences in risk classification may partly account for the observed differences in calibration and DCA between the two models.

Although the 11-point score was developed from a Japanese cohort, it tended to overestimate the LNM risk, which possibly reflected the differences in specimen types. This model was developed using surgical specimens, whereas the present study used endoscopic resection specimens. Surgical cohorts might include cases of deeper submucosal invasion or lesions not suitable for endoscopic treatment, resulting in a higher baseline LNM incidence compared with post-endoscopic resection populations. In addition, differences in pathological processing, such as sectioning intervals (typically 5 mm for surgical specimens vs. approximately 2 to 3 mm for endoscopic specimens), might influence the detection of relevant pathological features. These factors might have contributed to the discrepancies between the predicted and observed risks. Therefore, caution is required when applying this model to decision regarding additional gastrectomy following noncurative ESD. This limited applicability was reflected using the DCA, in which the 11-point score indicated a negative net benefit at lower threshold probabilities (starting at 0.06). By contrast, the eCura system and W-eCura score demonstrated positive net benefit across clinically relevant thresholds, ranging between 0.02 and 0.13 and between 0.02 and 0.10, respectively. The decline in the net benefit at higher thresholds possibly reflected the limited clinical relevance of such thresholds in routine practice. After recalibration, calibration improved across all models, and differences in net benefit were reduced, indicating that miscalibration was mainly attributable to differences in baseline risk rather than the model structure. Therefore, recalibration based on observed risks in the target population is essential when applying prediction models to different clinical settings.

Although discrimination was comparable across the models, the eCura system showed relatively favorable performance regarding calibration and potential clinical utility. Additionally, it offers the advantage of parsimony due to its limited number of predictors, which might facilitate clinical application when the predictive performance is similar [15–17]. Despite these advantages, the eCura system did not achieve sufficient calibration in the present study, possibly reflecting differences in the baseline LNM risk in our cohort. This is supported by the lower observed LNM incidence in our study, with an overall rate of 4.9%, which is lower than the approximately 8.0% reported in previous studies and was also lower in the high-risk group (13.0% vs. 22.7% in the original eCura study) [18]. One possible explanation for these differences is the higher rate of additional surgeries performed in the present study. Although the original eCura and W-eCura studies reported additional gastrectomy rates of approximately 55% and 52% among patients who underwent noncurative ESD, respectively, the rate in this study exceeded 84%, allowing pathological evaluation in a larger proportion of patients. This might have reduced selection bias and enabled a more accurate estimation of the LNM incidence. In addition, all the specimens were carefully re-evaluated by an expert gastrointestinal pathologist with additional immunohistochemical and special stains used as appropriate, which might have improved the detection of lymphatic and venous invasion.

Consequently, pathological risk factors such as lymphatic and venous invasion might have been more accurately detected. Only two cases were reclassified from noncurative to curative resection after pathological re-evaluation, suggesting that the potential impact of misclassification in the original pathological assessment on model validation was limited. Overall, this cohort, characterized by a high rate of additional surgery and detailed pathological evaluation, might better reflect the LNM risk in clinical practice. In the subgroup analysis according to the histological type, the eCura system maintained acceptable discrimination in the poor group, with c-statistic values of 0.75 for the 7-point score and 0.72 for the three-risk classification. A recent nationwide Japanese cohort study by Shiotsuki et al. also supported the applicability of the eCura system in patients with undifferentiated-type EGC. Our findings are consistent with this recent evidence and further support the potential applicability of the eCura system across the histological subgroups [19].

Given the moderate discrimination of the current models, further improvements are required. Tumor-derived biomarkers such as circulating tumor DNA, microRNA, and mRNA, as assessed by liquid biopsy, have been reported in other cancer types, and molecular alterations, including RhoGAP fusions, have been associated with LNM in gastric cancer [20–22]. These factors might further improve risk stratification.

This study has some limitations. First, this was a retrospective, single-center study, which might limit generalizability of the results. The rate of additional surgery in this cohort was higher than that in previous studies and might have reduced the bias in the observed incidence of LNM; however, the possibility of selection bias cannot be fully excluded. Moreover, because patients managed with surveillance were excluded owing to the lack of pathological confirmation of LNM, whether risk stratification using these models can reduce unnecessary surgery could not be evaluated. Second, this study was conducted in a Japanese cohort; thus, the findings may not be directly applicable to populations with different epidemiological and pathological backgrounds. Therefore, external validation in diverse populations is required. Third, the sample size for the external validation was not determined a priori. Although no consensus exists about sample size calculation for validation studies, adequate event numbers are important for reliable model evaluation [23]. In particular, the limited number of LNM events (n = 26) may have reduced the precision of the calibration estimates; hence, the observed differences in calibration performance should be interpreted with caution. Furthermore, the recalibrated models were evaluated in the same cohort used for recalibration, without internal validation. Consequently, the apparent improvement in calibration may be optimistic and requires validation in an independent cohort. Fourth, DCA estimates the theoretical net benefit and does not directly demonstrate improvement in actual patient outcomes. Finally, long-term clinical outcomes, including recurrence and survival, were not evaluated; therefore, the impact of model-based risk stratification on long-term patient outcomes could not be assessed. Despite these limitations, to our best knowledge, this study is among the first to comprehensively compare multiple LNM prediction models in this clinical setting and provide important insights into their performance and applicability.

In conclusion, although discrimination was similar across the models, the eCura system (both the 7-point score and three-risk classification) demonstrated relatively favorable calibration and potential clinical utility in this Japanese cohort. Given the structural similarity and comparable discrimination between the eCura system and W-eCura score, differences in performance could be possibly attributable to variations in the baseline risk rather than model specifications. Owing to their simplicity and favorable performance, the three-risk classification of the eCura system and W-eCura score, when appropriately calibrated, might offer a practical approach for routine decision-making regarding additional gastrectomy after noncurative ESD.

## Supplementary Information

Below is the link to the electronic supplementary material.

Supplementary file1 (DOCX 27 KB)

## Data Availability

The data underlying this article cannot be shared publicly because of institutional and ethical restrictions. De-identified data may be made available from the corresponding author upon reasonable request.
