# Supplementary material for: External validation and comparison of five prediction models for lymph node metastasis after noncurative endoscopic submucosal dissection for early gastric cancer
Source: Gastric Cancer. 2026 Sep 7;29(6):1306–15. doi: 10.1007/s10120-026-01798-9 (PMC13755012; doi:10.1007/s10120-026-01798-9)
Supplement: Supplementary file 1 [file 10120_2026_1798_MOESM1_ESM.docx]

**Supplementary Table 1: Scoring components and risk classification of prediction models for lymph node metastasis after noncurative ESD**

| **eCura system** | |
| --- | --- |
| **7-point score** | |
| **Variable** | **Point** |
| Lymphatic invasion | 3 |
| Tumor size >30 mm | 1 |
| Submucosal invasion ≥500 μm | 1 |
| Venous invasion | 1 |
| Positive vertical margin | 1 |
| **Three-risk classification based on total score** | |
| Low risk: 0–1 point | |
| Intermediate risk: 2–4 points | |
| High risk: 5–7 points | |
| **W- eCura score** | |
| **7-point score** | |
| **Variable** | **Point** |
| Lymphatic invasion | 3 |
| Tumor size >30 mm | 1 |
| Submucosal invasion ≥1000 μm | 1 |
| Venous invasion | 1 |
| Positive vertical margin | 1 |
| **Three-risk classification based on the total score** | |
| Low risk: 0–1 point | |
| Intermediate risk: 2–4 points | |
| High risk: 5–7 points | |
| **11-point score** | |
| **Variable** | **Point** |
| Lymphovascular invasion | 4 |
| Tumor size |  |
| >20 mm and ≤30 mm | 1 |
| >30 mm | 2 |
| Submucosal invasion ≥500 μm | 2 |
| Histological type |  |
| Pure undifferentiated-type (poor) | 1 |
| Mixed type, differentiated-type (well/moderate) predominant | 2 |
| Mixed type, undifferentiated-type (poor) predominant | 2 |
| Ulcerative findings | 1 |

The eCura system and the W-eCura score assign points based on the pathological findings of endoscopic resection specimens. The W-eCura score differs from the eCura system in the cutoff value for submucosal invasion depth (≥1000 μm vs ≥500 μm). In the 11-point scale, the histological type includes pure differentiated (pure well/moderate), pure undifferentiated (pure poor), and mixed types; points are assigned to pure undifferentiated (pure poor) and mixed types, while the pure differentiated type (well/moderate) is assigned 0 points.

ESD, endoscopic submucosal dissection.

**Supplementary Table 2: Distribution of the LNM rates in the original and present cohorts across the five prediction models**

| **Model** | **Category** | **Original LNM, % (n/N)** | **Observed LNM in the present study, % (n/N, 95% CI)** |
| --- | --- | --- | --- |
| eCura system | 0 | 1.6% (1/62) | 1.9% (2/104; 0.2 to 6.8) |
| (7-point score) | 1 | 2.6% (9/341) | 1.4% (2/140; 0.2 to 5.1) |
|  | 2 | 4.9% (9/185) | 1.5% (1/68; 0.0 to 7.9) |
|  | 3 | 7.4% (11/148) | 9.0% (7/78; 3.7 to 17.6) |
|  | 4 | 8.3% (11/132) | 5.9% (4/68; 1.6 to 14.4) |
|  | 5 | 19.9% (28/141) | 13.0% (7/54; 5.4 to 24.9) |
|  | 6 | 27.3% (21/77) | 9.1% (2/22; 1.1 to 29.2) |
|  | 7 | 26.7% (4/15) | 100% (1/1; 2.5 to 100) |
| eCura system | Low | 2.5% (10/403) | 1.6% (4/244; 0.4 to 4.1) |
| (three-risk classification) | Intermediate | 6.7% (31/465) | 5.6% (12/214; 2.9 to 9.6) |
|  | High | 22.7% (53/233) | 13.0% (10/77; 6.4 to 22.6) |
| W-eCura score | 0 | 0% (0/21) | 1.4% (2/138; 0.2 to 5.1) |
| (7-point score) | 1 | 0% (0/42) | 1.4% (2/144; 0.2 to 4.9) |
|  | 2 | 0% (0/20) | 9.4% (3/32; 2.0 to 25.0) |
|  | 3 | 8.3% (2/24) | 6.5% (6/93; 2.4 to 13.5) |
|  | 4 | 20.0% (4/20) | 7.9% (6/76; 3.0 to 16.4) |
|  | 5 | 57.1% (12/21) | 11.4% (4/35; 3.2 to 26.7) |
|  | 6 | 71.4% (5/7) | 12.5% (2/16; 1.5 to 38.4) |
|  | 7 | 0% (0/1) | 100% (1/1; 2.5 to 100) |
| W-eCura score | Low | 0% (0/63) | 1.4% (4/282; 0.4 to 3.6) |
| (three-risk classification) | Intermediate | 9.4% (6/64) | 7.5% (15/201; 4.2 to 12.0) |
|  | High | 58.6% (17/29) | 13.5% (7/52; 5.6 to 25.8) |
| 11-point score | 0 | 0.0% (0/67) | 0.0% (0/2; 0.0 to 84.2) |
|  | 1 | 0.7% (2/298) | 4.3% (1/23; 0.1 to 21.9) |
|  | 2 | 1.8% (9/493) | 1.5% (1/67; 0.0 to 8.0) |
|  | 3 | 3.7% (23/621) | 1.3 % (1/78; 0.0 to 6.9) |
|  | 4 | 7.9% (46/585) | 1.2% (1/84; 0.0 to 6.5) |
|  | 5 | 12.6% (38/302) | 3.6% (2/55; 0.4 to 12.5) |
|  | 6 | 17.2% (36/209) | 6.2% (4/65; 1.7 to 15.0) |
|  | 7 | 26.0% (34/131) | 8.1% (6/74; 3.0 to 16.8) |
|  | 8 | 35.1% (54/154) | 9.3% (5/54; 3.1 to 20.3) |
|  | 9 | 49.0% (72/147) | 14.8% (4/27; 4.2 to 33.7) |
|  | 10 | 56.1% (55/98) | 16.7% (1/6; 0.4 to 64.1) |
|  | 11 | 65.4% (17/26) | - |

Values represent the number of patients with LNM and the corresponding LNM rates with 95% CI in the present cohort. Original LNM rates were extracted from original publications for each risk model. For the W-eCura (7-point score), category-specific data were obtained through personal communication with the original author.

LNM, lymph node metastasis; CI, confidence interval.

**Supplementary Table 3: Risk classification and lymph node metastasis rates in patients with submucosal invasion depth of 500 to 999 μm**

| **eCura system** | **W-eCura score** | **Patients, n** | **LNM, n (%)** |
| --- | --- | --- | --- |
| Low | Low | 31 | 0 (0) |
| Intermediate | Low | 38 | 0 (0) |
| Intermediate | Intermediate | 20 | 3 (15.0) |
| High | Intermediate | 25 | 3 (12.0) |
| High | High | 6 | 0 (0) |
| **Total** |  | **120** | **6 (5.0)** |

Values represent the distribution of patients and corresponding LNM rates for each combination of risk categories in the eCura system and W-eCura score. Risk categories were defined as low (0–1 point), intermediate (2–4 points), and high (5–7 points) for both models.

LNM, lymph node metastasis.

**Supplementary Table 4: Performance of the five prediction models after recalibration of both the intercept and the slope for predicting LNM in patients with additional surgery after noncurative ESD**

|  | Calibration-in-the-large | Calibration slope | Brier score |
| --- | --- | --- | --- |
| eCura system (7-point score) | 0.00 (−0.40 to 0.40) | 1.00 (0.50 to 1.50) | 0.05 (0.03 to 0.06) |
| eCura system (three-risk classification) | 0.00 (−0.40 to 0.40) | 1.00 (0.49 to 1.51) | 0.04 (0.03 to 0.06) |
| W-eCura score (7-pont score) | 0.00 (−0.40 to 0.40) | 1.00 (0.44 to 1.56) | 0.04 (0.03 to 0.06) |
| W-eCura score (three-risk classification) | 0.00 (−0.40 to 0.40) | 1.00 (0.50 to 1.50) | 0.04 (0.03 to 0.06) |
| 11-point score | 0.00 (−0.40 to 0.40) | 1.00 (0.41 to 1.59) | 0.04 (0.03 to 0.06) |

Values are presented as calibration-in-the-large, calibration slope, and Brier score with 95% CIs. Calibration-in-the-large reflects the systematic over- or under-estimation of the predicted risk, with values close to zero indicating good calibration. The calibration slope assessed the agreement between the predicted and observed risks, with a value of 1 indicating perfect calibration. Lower Brier scores indicated better overall prediction accuracy.

LNM, lymph node metastasis; ESD, endoscopic submucosal dissection; CI, confidence interval
